# Supplementary material for: Chromothripsis during telomere crisis is independent of NHEJ, and consistent with a replicative origin
Source: Genome Res. 2019 May;29(5):737–49. doi: 10.1101/gr.240705.118 (PMC6499312; doi:10.1101/gr.240705.118)
Supplement: Supplemental Material [file supp_gr.240705.118_Supplemental_file_1.zip › contigs/annotated_contigs/DB40/contig.2.DB40_length_337_mean_cov_4.0059347181.docx]

**DB40_length_337_mean_cov_4.0059347181**

```
GAGACACATCATTCATTGCTCCTACTTGTGTCTGTAATCAGAGAGTATTTAAGGAGGGCAGTTCTGCGACGGACTGTGTGTGTGTTGAA
 >chr10:98103989-98104106 - E=5e-59 p=1e-03
AGTAAAGATGGGGGGCCGCGGGGAA|GGC|TCTAGAATCATTATAAGACAGAGGGTGGGGCGTTCCAAAGAGGCAAAGATCAATCGTGT
                          >chr10:98166902-98167125 - E=5e-123
TCATCAGGGCAATCAGACACAGGCTCGAGGAGGGGGACACTTGAGCAGGCCTGGGAAGGGTTTCTCTGACATGGGACTGTCAGGGAACA

CTGGGAAACAGGTTGCACAGGACAGGCTCTGGGGGTGTGGAAAGTAAGCAGAATTTAATTCATGAGATGGGG
```
